# Root Bark of *Morus alba* L. and Its Bioactive Ingredient, Ursolic Acid, Suppress the Proliferation of Multiple Myeloma Cells by Inhibiting Wnt/β-Catenin Pathway

**DOI:** 10.4014/jmb.2109.09002

**Published:** 2021-09-25

**Authors:** Geu Rim Song, Yoon Jung Choi, Soo Jin Park, Subeen Shin, Giseong Lee, Hui Ji Choi, Do Yup Lee, Gyu-Yong Song, Sangtaek Oh

**Affiliations:** 1 Department of Bio and Fermentation Convergence Technology, Kookmin University, Seoul 02707, Republic of Korea; 2 College of Pharmacy, Chungnam National University, Daejeon 34134, Republic of Korea; 3 Department of Agricultural Biotechnology, Center for Food and Bioconvergence, Research Institute for Agricultural and Life Sciences, Seoul National University, Seoul 08826, Republic of Korea; 4 Department of Interdisciplinary Program for Bio-Health Convergence, Kookmin University, Seoul 02707, Republic of Korea; 5 College of General Education, Kookmin University, Seoul 02707, Republic of Korea

**Keywords:** *Morus alba* L., Wnt/β-catenin signaling, multiple myeloma, ursolic acid

## Abstract

The root bark of *Morus alba* L. has cytotoxic activity against several types of cancer cells. However, little is known about its chemopreventive mechanisms and bioactive metabolites. In this study, we showed that *M. alba* L. root bark extracts (MRBE) suppressed β-catenin response transcription (CRT), which is aberrantly activated in various cancers, by promoting the degradation of β-catenin. In addition, MRBE repressed the expression of the β-catenin/T-cell factor (TCF)-dependent genes, cmyc and cyclin D1, thus inhibiting the proliferation of RPMI-8226 multiple myeloma (MM) cells. MRBE induced apoptosis in MM cells, as evidenced by the increase in the population of annexin VFITC- positive cells and caspase-3/7 activity. We identified ursolic acid in MRBE through LC/mass spectrum (MS) and observed that it also decreased intracellular β-catenin, c-myc, and cyclin D1 levels. Furthermore, it suppressed the proliferation of RPMI-8226 cells by stimulating cell cycle arrest and apoptosis. These findings suggest that MRBE and its active ingredient, ursolic acid, exert antiproliferative activity by promoting the degradation of β-catenin and may have significant chemopreventive potential against MM.

## Introduction

Multiple myeloma (MM), a malignant plasma cell neoplasm, is the second most frequent hematological malignancy, comprising 10–15% of all blood cancers [1]. Well-established MM characteristics include the infiltration and clonal expansion of plasma cells within the bone marrow and the excessive accumulation of serum immunoglobulin [2]. Because MM is not currently curable, developing new therapeutics based on the underlying molecular mechanisms altered in these cancer cells is urgently needed.

The excessive accumulation of β-catenin and the subsequent upregulation of β-catenin response transcription (CRT) contribute to the development and progression of several cancers [3]. In MM, the Wnt/β-catenin pathway is constitutively activated, and the intracellular β-catenin levels are elevated [4]. β-catenin translocates to the nucleus, where it forms a complex with T-cell factor (TCF)/lymphocyte enhancer factor family transcription factors (LEF). This complex activates the expression of target genes that play essential roles in tumor development, including c-myc, cyclin D1, metalloproteinase-7, and peroxisome proliferator-activated receptor-δ [5-8]. Therefore, accelerating β-catenin turnover may be an effective strategy for chemoprevention and treatment of MM.

The root bark of *Morus alba* L. has been used in herbal medicine because of its anti-inflammatory, liver-protective, kidney-protective, hypotensive, diuretic, anti-cough, and analgesic effects [9]. Recently, *M. alba* L. root bark extracts (MRBE) were shown to have anticancer activity [10, 11] and suppress the migration of human non-small-cell lung cancer cells [12]. However, the molecular mechanisms behind this potential anticancer activity and the active ingredients responsible have not been elucidated. In this study, we found that MRBE inhibit CRT-positive cancer cell proliferation by inhibiting the Wnt/β-catenin pathway. We also identified ursolic acid as the bioactive ingredient underlying this activity.

## Materials and Methods

### Preparation of *M. alba* L. Root Bark Extract

The dried root barks of *M. alba* L. (1 kg) were extracted with 80% (v/v) ethanol (3 L) under reflux 3 times for 2 h. After the liquid was filtered, the fluid was concentrated under vacuum to obtain the extracts (9.7 g).

### Cell Cultures, Reporter Assays, and Chemicals

RPMI8226, HEK293, and Wnt3a-L cells were purchased from the American Type Culture Collection and cultured in RPMI1640 (RPMI8226 and MM.1S) and Dulbecco’s modified Eagle’s media (HEK293 and Wnt3a-L) supplemented with 10% fetal bovine serum, 120 μg/ml penicillin, and 200 μg/ml streptomycin. HEK293-FL (TOPFlash) reporter cells were established as previously described [13]. Wnt3a-CM was prepared as previously described [14]. Luciferase assays were conducted using the Dual-Luciferase Assay Kit (Promega, USA), following the manufacturer’s instructions. Lithium chloride (LiCl), MG-132, and ursolic acid were purchased from Sigma-Aldrich (USA).

### Western Blot Assays

Cytosolic fractions were obtained as previously described [15]. Whole-cell fractions were prepared in RIPA buffer. We used the Bradford assay for protein quantization. Proteins (30 μg) were separated by SDS-PAGE in 4–12% gradient gels (Invitrogen, USA) and transferred to nitrocellulose membranes (Bio-Rad Laboratories, USA). The membranes were soaked in SuperBlock Blocking Buffer (Thermo Scientific) for 1 h at room temperature and then incubated overnight with primary antibodies. The following primary antibodies were used: anti-β-catenin (BD Transduction Laboratories, USA), anti-cyclin D1 (Santa Cruz Biotechnology, USA), anti-c-myc (Santa Cruz Biotechnology), anti-poly(ADP) ribose polymerase (anti-PARP; Cell Signaling Technology, USA), anti-cleaved caspase-3 (Cell Signaling Technology), anti-phospho-β-catenin (Ser33/37/Thr41) (Cell Signaling Technology) and anti-actin (Cell Signaling Technology). The membranes were then incubated with horseradish peroxidase-conjugated anti-mouse IgG (Santa Cruz Biotechnology) or anti-rabbit IgG (Santa Cruz Biotechnology). The bands were visualized using the ECL system (Santa Cruz Biotechnology) and quantified using the ImageJ software (NIH, USA).

### RNA Isolation and Real-Time PCR

Total RNA was isolated using Trizol reagent (Invitrogen) following the manufacturer’s protocol. cDNA synthesis and PCR were performed as previously described [14]. The amplified DNA was separated using 2%agarose gels and stained with LoadingStar (Dynebio, Korea). For real-time PCR, the CFX96 qPCR system (Bio-Rad) was used. Reactions were carried out in 96-well optical reaction plates in a 20 μl final volume containing 10 μl of the 2X SsoFast EvaGreen Supermixes (Bio-Rad), 1 μl of each gene-specific forward primer, 1 μl of each gene-specific reverse primer, 1 μl of diluted cDNA sample, and 7 μl of water. After an initial denaturing step for 10 min at 95°C, conditions for cycling were set to 40 cycles of 30 s at 94°C, 30 s at 58°C, and 30 s at 72°C. The changes in gene expression were quantified by the comparative Ct method by calculating the relative fold changes normalized against β-actin expression [16].

### Cell Viability Assays

Cells were inoculated into 96-well plates and treated with either DMSO or MRBE (20 and 40 μg/mL) and ursolic acid (20 and 40 μM). After 48 h, the cell viability of each treated sample was measured in triplicate with the Celltiter-Glo Assay Kit (Promega) according to the manufacturer’s instructions. To calculate the inhibition of cell growth, the value at time 0 was subtracted.

### Apoptosis Analysis

After treatment with either DMSO or MRBE (20 and 40 μg/ml) and ursolic acid (20 and 40 μM) for 48 h, cells were washed with cold phosphate-buffered saline and stained with annexin V-FITC and PI. The levels of apoptosis were then measured using the Apoptosis Detection Kit (BD Transduction Laboratories), according to the manufacturer’s protocols. Cells were then analyzed using a Cellometer cytometer (Nexcelom, USA).

### Metabolite Profiling by UPLC-Orbitrap MS

*M. alba* L. root bark metabolites were extracted as described previously [17, 18]. An Ultimate-3000 UPLC system (Thermo Fisher Scientific, USA) coupled with a Q-Exactive Focus Hybrid Quadrupole-Orbitrap Mass Spectrometer Plus instrument (Thermo Fisher Scientific) was used to perform the LC-MS/MS analysis. The metabolite separation from MRBE (20 mg) was conducted on a UPLC BEH C_18_ 1.7 μm 2.1 × 150mm column (Waters, USA) equipped with UPLC BEH C_18_ 1.7 μm 2.1 × 5.0 mm VanGuard Pre-Column (Waters). The mobile phase consisted of 0.1% formic acid in water (buffer A) and 0.1% formic acid in acetonitrile (buffer B). The chromatographic separation was programmed as follows (buffer B): 0–2.0 min, 5%; 2.0–25.0 min, 5–95%; 25.0–27.5 min, 95%; and 27.6–30.0 min, 5%. The flow rate was set to 300 μL/min. The mass spectra data were acquired using Full MS-ddMS2 (top3) scan mode in positive ionization mode. Data acquisition and processing were performed using the Xcalibur software (Thermo Fisher Scientific).

### Caspase 3/7 Assay

The activity of caspase-3/7 was determined in cell lysates using the Apo-ONE Homogeneous Caspase-3/7 Assay Kit (Promega).

### Statistical Analysis

Student's *t*-tests were employed to compare the means of the control and experimental groups. All experiments were conducted three times. Statistical significance was set to *p* < 0.05 (*) or *p* < 0.01 (**). The results were expressed as mean ± standard deviation (SD).

## Results

### MRBE Inhibits the Wnt/β-Catenin Pathway by Promoting GSK-3β-Independent β-Catenin Degradation

**Fig. 1:**
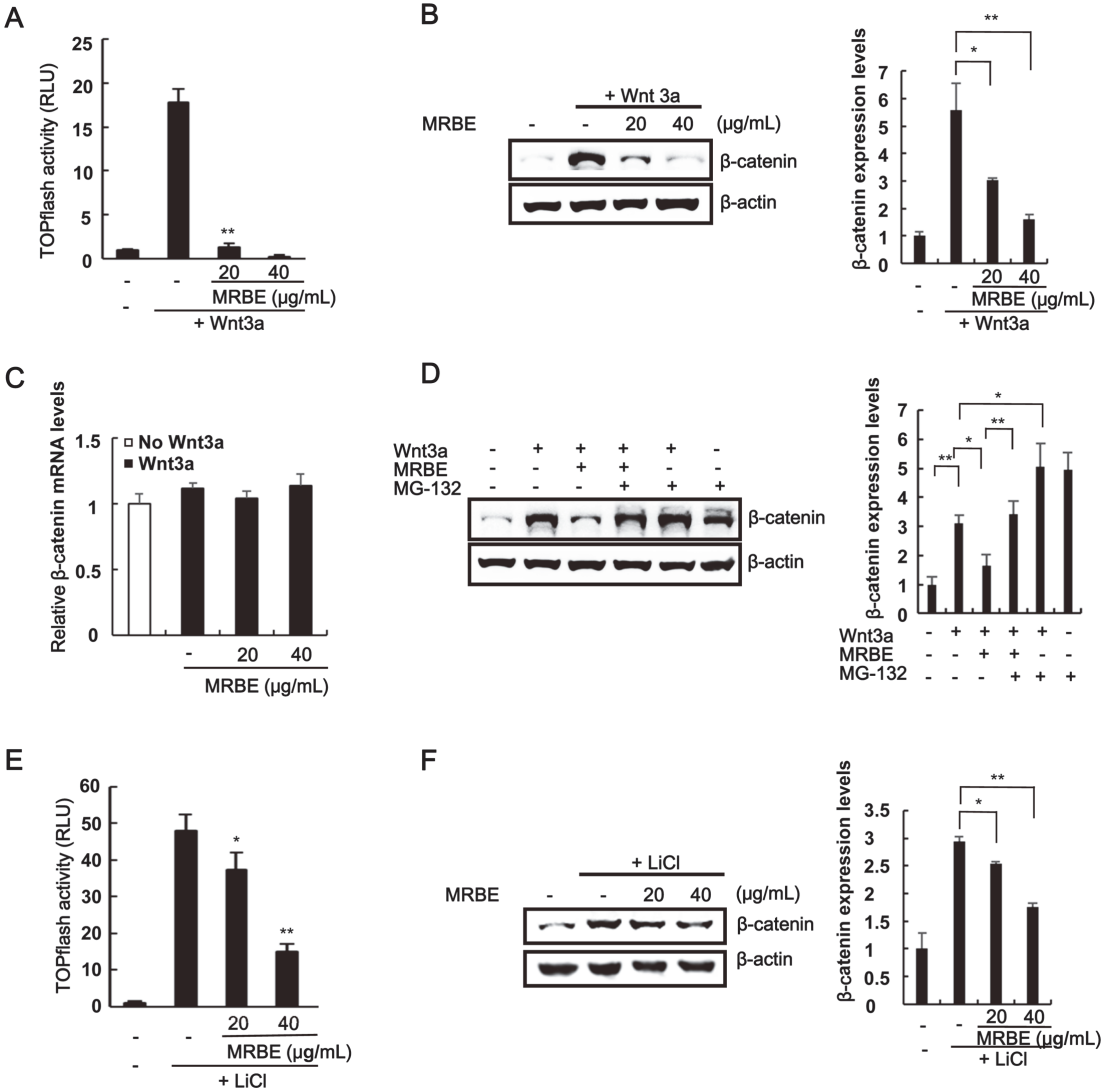
Identification of *Morus alba* L. root bark extract (MRBE) as an inhibitor of the Wnt/β-catenin pathway. (**A**) Firefly luciferase (FL) activity determined in HEK293-FL reporter cells incubated with either DMSO (vehicle) or MRBE (20 and 40 μg/ml) in the presence of Wnt3a-CM for 15 h. (**B**) Downregulation of β-catenin protein levels by MRBE in the presence of Wnt3a-CM. (**C**) Effect of MRBE on β-catenin mRNA levels. Real-time PCRs for the analysis of β-catenin and GAPDH were performed with total RNA prepared from HEK293-FL reporter cells treated with either DMSO or the indicated concentrations of MRBE, in the presence of Wnt3a-CM, for 15 h. (**D**) Western blot with the anti-β-catenin antibody on cytosolic proteins prepared from HEK293-FL reporter cells exposed to MRBE (20 μg/ml) for 15 h and MG-132 (10 μM) for 8 h. (**E**) Luciferase assay of HEK293 reporter cells incubated with MRBE in the presence of 20 mM LiCl for 15 h. (**F**) Western blot with the β-catenin antibody on cytosolic proteins from HEK293 reporter cells treated with DMSO or increasing amounts of MRBE in the presence of 40 mM LiCl for 15 h. The results represent the mean ± SD of three independent experiments. *p < 0.05 and **p < 0.01, cells treated with either Wnt3a-CM or LiCl compared with cells treated with MRBE.

To elucidate the mechanisms underlying the MRBE anticancer activity, we used HEK293-firefly luciferase (FL) reporter cells stably harboring a synthetic β-catenin/TCF-dependent FL reporter (TOPFlash) and an hFz-1 expression plasmid. The incubation of HEK293-FL reporter cells with Wnt3a-conditioned medium (Wnt3a-CM) increased CRT activity. However, MRBE addition led to a concentration-dependent decrease in CRT (Fig. 1A). We then employed western blot analysis to test the effects of MRBE on intracellular levels of β-catenin, a principal regulator of CRT in the Wnt/β-catenin pathway. As shown in Fig. 1B, MRBE reduced the cytoplasmic β-catenin protein levels accumulated by Wnt3a-CM in HEK293-FL reporter cells. Under these conditions, β-catenin mRNA levels were not altered by MRBE (Fig. 1C). β-Catenin is targeted by a proteasome-dependent degradation pathway [19]; hence, we used the proteasome inhibitor MG-132 to examine the involvement of the proteasome in β-catenin downregulation by MRBE. As depicted in Fig. 1D, the treatment of HEK293-FL reporter cells with MRBE consistently decreased intracellular β-catenin levels, but the addition of MG-132 abolished this effect. Taken together, these data indicate that MRBE suppresses the Wnt/β-catenin pathway by inducing proteasome-dependent β-catenin degradation.

Glycogen synthase kinase-3β (GSK-3β)-mediated β-catenin phosphorylation at Ser33/37/Thr41 is central in the proteasome-dependent β-catenin degradation pathway [20]. Therefore, we examined whether GSK-3β activity is required for MRBE-induced β-catenin degradation. As shown in Fig. 1E, the treatment of HEK293-FL reporter cells with MRBE still led to a dose-dependent decrease in CRT in the presence of LiCl, which is a GSK-3β inhibitor that increases CRT [21]. Moreover, western blot analysis using an anti-β-catenin antibody showed that MRBE treatments reduced the intracellular β-catenin levels increased by LiCl (Fig. 1F). Next, we examined whether MRBE induces the Ser33/37/Thr41 phosphorylation of β-catenin by western blot analysis with a phospho-specific β-catenin antibody, In the presence of LiCl, the β-catenin phosphorylation levels at Ser33/37/Thr41 residues were decreased but were increased with the addition of MRBE (Fig. S1). Taken together, these results suggest that MRBE can suppress the Wnt/β-catenin pathway partly by inducing GSK-3β-independent β-catenin degradation.

### MRBE Inhibits MM Cell Proliferation by Downregulating β-Catenin Levels

**Fig. 2:**
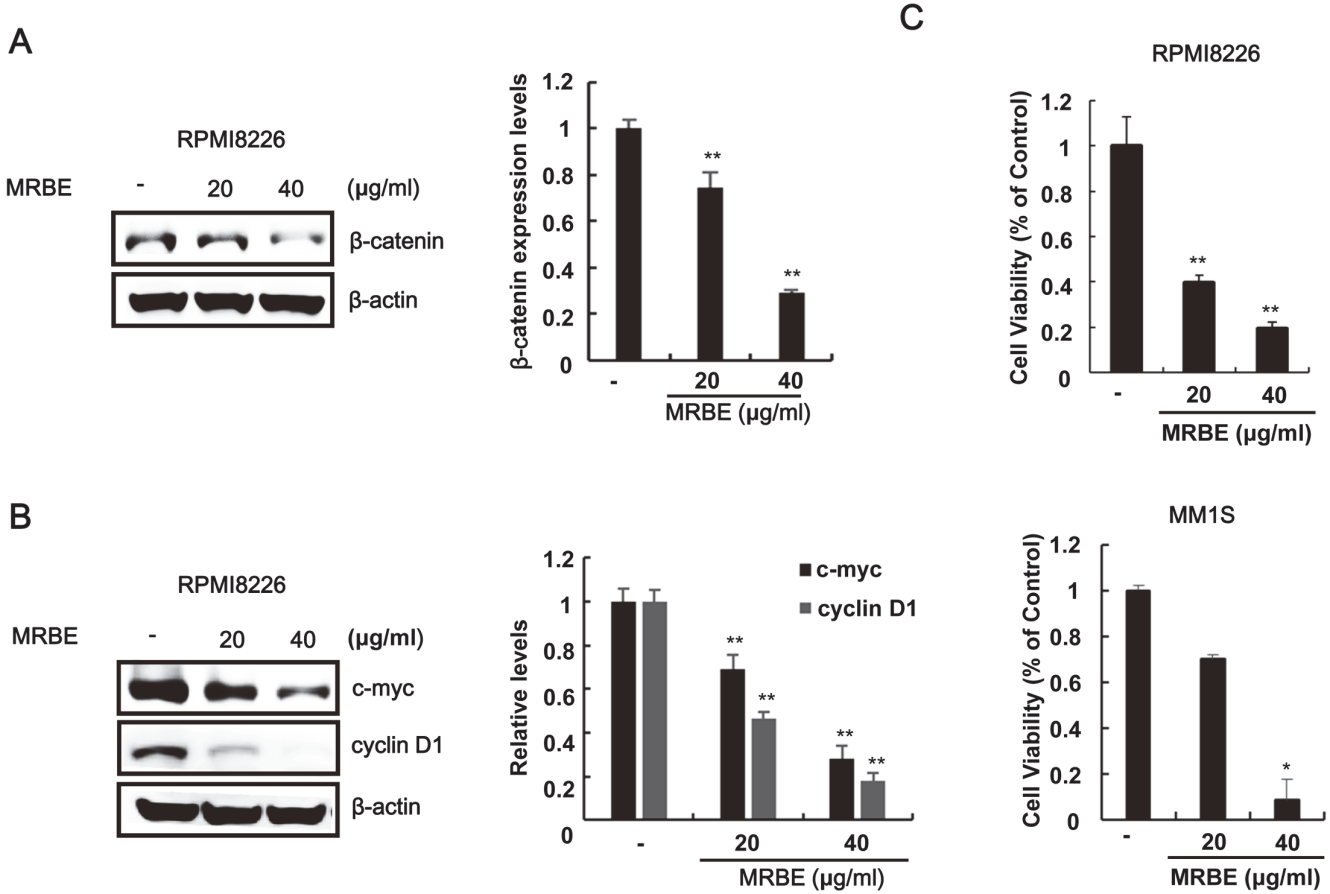
MRBE inhibits the expression of β-catenin and its dependent genes in RPMI8226. (**A**) Effect of MRBE on β-catenin protein levels in RPMI8226 cells. (**B**) Western blot with anti-c-myc, anti-cyclin D1, and anti-β-actin (loading control) antibodies on extracts of RPMI-8226 cells incubated with either DMSO (vehicle) or MRBE (20 and 40 μg/ml) for 15 h. (**C**) Cell viability of RPMI-8226 cells and MM.1S. Cells were incubated for 48 h in 96-well plates. To calculate the inhibition of cell growth, the value at time 0 was subtracted. The results represent the mean ± SD of three independent experiments. *p < 0.05 and **p < 0.01, compared with the DMSO control.

A previous study demonstrated that β-catenin is constitutively upregulated in RPMI-8226 and MM.1S MM cells [22]. We investigated the effect of MRBE treatments on β-catenin levels in RPMI-8226 cells through western blot analysis with an anti-β-catenin antibody. As presented in Fig. 2A, MRBE decreased intracellular β-catenin levels in RPMI-8226 cells. We then examined the effect of different MRBE concentrations on the expression of β-catenin target genes in RPMI-8226 cells through western blot analysis. MRBE caused a dose-dependent downregulation of the well-characterized β-catenin-dependent genes, c-myc and cyclin D1 (Fig. 2B). Because the disruption of β-catenin function can inhibit the proliferation of MM cells [23], we assessed the effect of MRBE on the growth of MM cells. As depicted in Fig. 2C, MRBE efficiently inhibited the proliferation of RPMI-8226 and MM.1S cells in a concentration-dependent manner. These results suggest that MRBE suppresses the proliferation of MM cells by downregulating β-catenin levels.

### MRBE Induces Apoptosis in RPMI-8226 MM Cells

**Fig. 3:**
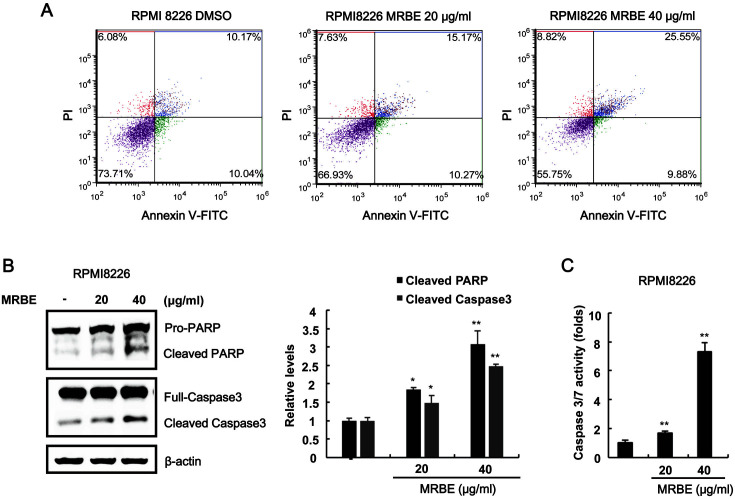
Apoptotic effects of MRBE on RPMI-8226 cell growth. (**A**) Cytometry analysis of RPMI-8226 cells incubated with either DMSO (vehicle) or MRBE (20 and 40 μg/ml) for 48 h. After incubation, cells were harvested and stained with annexin V-FITC and propidium iodide (PI). The x-axis indicates the annexin V-FITC intensity, while the y-axis indicates the PI fluorescence. (**B**) Western blot with anti-caspase-3, anti-poly (ADP-ribose) polymerase (PARP), and anti-actin (loading control) antibodies on whole-cell extracts. The results are representative of three independent experiments. (**C**) Caspase-3/7 activity determined after RPMI8226 cell incubation with either DMSO or MRBE (20 and 40 μg/ml) for 48 h. The results represent the mean ± SD of three independent experiments. *p < 0.05 and **p < 0.01 compared with the DMSO control group.

To elucidate the antiproliferative mechanisms triggered by MRBE, we exposed RPMI-8226 cells to MRBE and determined the number of annexin V-FITC/propidium iodide-stained apoptotic cells using a cytometer. As presented in Fig. 3A, the population of apoptotic cells (annexinV-FITC/propidium iodide (PI) double-positive cells) increased in a dose-dependent manner. Western blot analysis showed that MRBE treatments led to the formation of active caspase-3 (cleaved form), which in turn catalyzed the cleavage of anti-poly (ADP-ribose) polymerase (PARP), a biochemical apoptosis marker (Fig. 3B). Consistently, the activity of caspase-3/7 increased in response to MRBE treatments (Fig. 3C). These results indicate that apoptosis contributes to the MRBE-induced RPMI-8226 cell growth inhibition.

### Identification of Ursolic Acid from MRBE through LC/Mass Spectrum (MS) Analysis

**Fig. 4:**
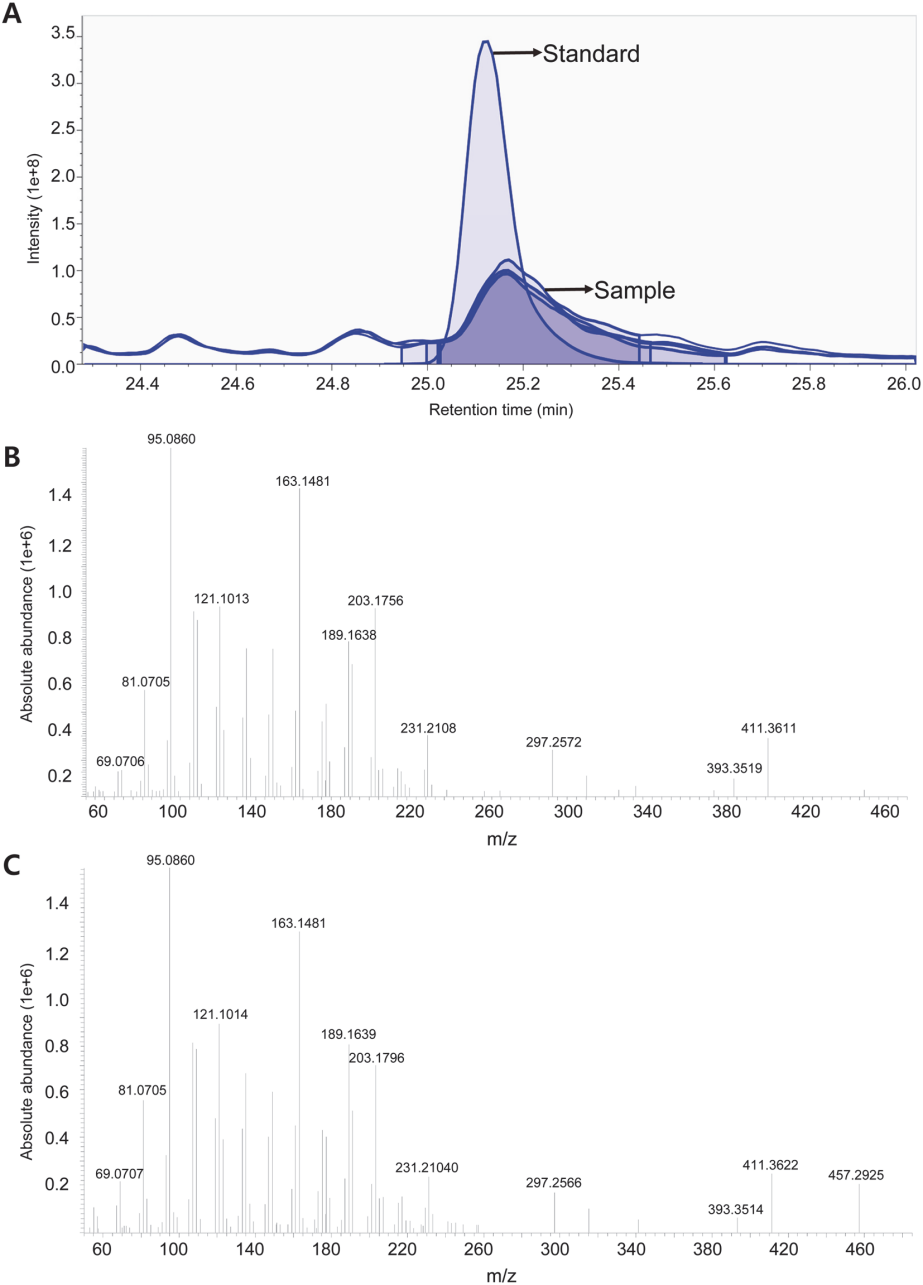
LC-MS analysis of ursolic acid in MRBE. (**A**) Extracted ion chromatogram of ursolic acid in standard solution and MRBE. The tandem mass spectra of ursolic acid in standard solution (**B**) and MRBE (**C**).

Secondary metabolites have been identified in the root bark of *M. alba* L., including terpenoids, flavonoids, and stilenoids [24]. To comprehensively profile the metabolite contents in the root bark extract (MRBE), we performed UPLC-Orbitrap MS analysis in an untargeted manner. A total of 121 metabolites were structurally identified and classified as amino acids, isoflavones, and isoflavonoids (Table S1). Among metabolites, ursolic acid is a pentacyclic terpenoid with a wide range of pharmaceutical properties found in the root bark of *M. alba* L.[24, 25]. The terpenoid has previously been demonstrated to inhibit the Wnt/β-catenin pathway and suppress the proliferation of adenomatous polyposis coli (APC)-mutated colon cancer, osteosarcoma, and PC-3 prostate cancer cells [26-28]. Accordingly, we determined ursolic acid in MRBE. The comparative analysis using the authentic compound of ursolic acid confirmed the base peak at 457.3674 m/z (25 min) as [M+H]^+^ ions (Fig. 4A). Furthermore, the tandem MS/MS spectrum of the ion matched the fragmentation pattern of the reference compound (Figs. 4B, C). Based on one-point calibration, the ursolic acid content was estimated as 28.96 µg in 20 mg of MRBE.

### Ursolic Acid Downregulates β-Catenin and Represses Its Target Genes in MM Cells

**Fig. 5:**
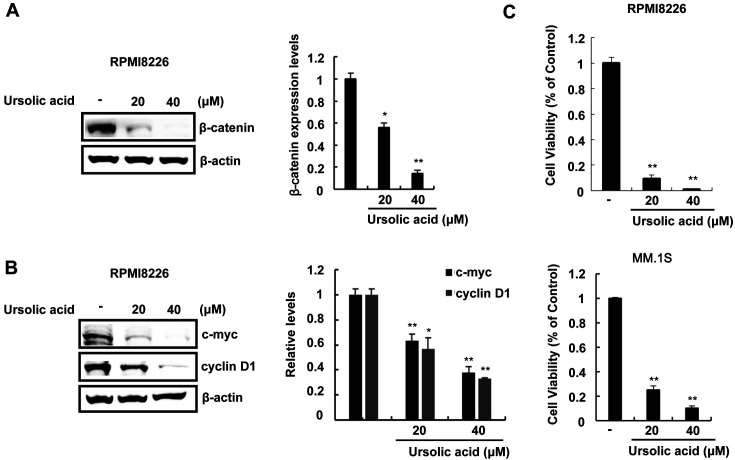
Ursolic acid inhibits the expression of β-catenin and its dependent genes in RPMI8226. (**A**) Effect of ursolic acid on β-catenin protein levels in RPMI8226 cells. (**B**) Western blot with anti-c-myc, anti-cyclin D1, and anti-β-actin (loading control) antibodies on extracts of RPMI-8226 cells incubated with the DMSO (vehicle) or ursolic acid (20 and 40 μM) for 15 h. (**C**) Cell viability of RPMI-8226 cells with the given concentrations of ursolic acid. RPMI-8226 cells were incubated for 48 h in 96-well plates. To calculate the inhibition of cell growth, the value at time 0 was subtracted. The results represent the mean ± SD of three independent experiments. *p < 0.05 and **p < 0.01, compared with the DMSO control group.

We then explored the effect of ursolic acid on MM. The incubation of RPMI-8226 and MM.1S cells with ursolic acid resulted in the downregulation of cytoplasmic β-catenin expression (Figs. 5A, S2). The effect of ursolic acid on the expression of β-catenin-dependent genes in RPMI-8226 cells was then evaluated. After treatment of RPMI-8226 cells with various amounts of ursolic acid, the expression levels of c-myc and cyclin D1 were measured using western blot analysis. As expected, we found a significant downregulation in c-myc and cyclin D1 protein levels (Fig. 5B). Consistent with the results from MRBE, ursolic acid efficiently suppressed the growth of RPMI-8226 and MM.1S cells (Fig. 5C). These results indicate that ursolic acid may be an active ingredient responsible for the MRBE-mediated inhibition of the Wnt/β-catenin pathway and MRBE-induced growth suppression in MM cells.

### Ursolic Acid Induces Cell Cycle Arrest and Apoptosis in RPMI-8226 MM Cells

**Fig. 6:**
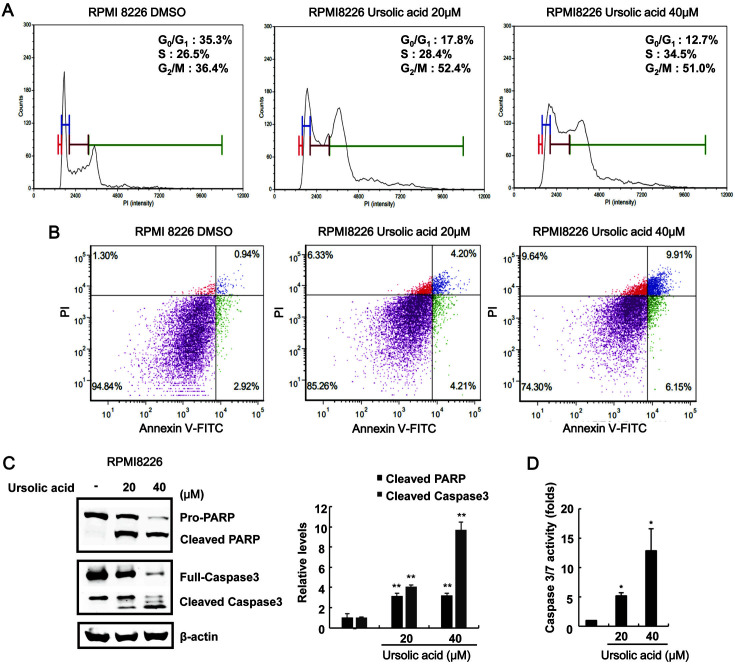
Apoptotic effects of ursolic acid on RPMI-8226 cell growth. (**A**) RPMI-8226 cells were incubated with the DMSO (vehicle) or ursolic acid (20 and 40 μM) for 48 h. After incubation, cells were harvested and stained with propidium iodide (PI) and analyzed using a cytometer. The x-axis indicates the PI fluorescence intensity that correlated with the DNA content. (**B**) RPMI-8226 cells were incubated with the DMSO (vehicle) or ursolic acid (20 and 40 μM) for 48 h. After incubation, cells were harvested and stained with annexin V-FITC and PI and analyzed using a cytometer. The x-axis indicates the annexin V-FITC intensity, while the y-axis indicates the PI fluorescence. (**C**) Western blot with anti-caspase-3, anti-poly (ADP-ribose) polymerase (PARP), and anti-actin (loading control) antibodies on whole-cell extracts. The results are representative of three independent experiments. (**D**) Caspase-3/7 activity determined after RPMI8226 cell incubation with either DMSO (vehicle) or ursolic acid (20 and 40 μM) for 48 h. The results represent the mean ± SD of three independent experiments. *p < 0.05 compared with the DMSO control group.

To investigate the possible mechanism for ursolic acid-mediated growth inhibition in MM cells, RPMI-8226 cells were treated with ursolic acid and the distribution of the cell cycle was examined after cell staining with propidium iodide (PI). As shown in Fig. S2, cytometric analysis demonstrated that the populations of RPMI-8226 in G2/M phase were elevated from 36.4 to 52.4%, compared with the vehicle control. We also analyzed the ability of ursolic acid to induce apoptosis in RPMI-8226 cells. Annexin V-FITC/PI staining showed that the percentage of apoptotic cells increased following ursolic acid treatment (Fig. 6B). In addition, incubating RPMI-8226 cells with ursolic acid stimulated the formation of cleaved caspase-3 and PARP forms (Fig. 6C) and increased caspase-3/7 activity (Fig. 6D). These results suggest that both cell cycle arrest at G2/M phase and apoptosis contributed to the ursolic acid-induced inhibition of MM cell proliferation.

## Discussion

Medicinal plants are potential resources for developing therapeutics targeting human diseases. The root bark of *M. alba* L., a traditional Chinese medicine, produces various natural compounds, including terpenoids, flavonoids, and stilenoids [24]. Interestingly, a recent study reported that MRBE has cytotoxic effects on non-small-cell lung cancer cells [11, 12]. However, the underlying antiproliferative mechanisms and the active metabolites associated remain unknown. In this study, we demonstrated that MRBE and its active ingredient, ursolic acid, are bona fide cancer antagonists as evidenced by their effect in downregulating CRT and cytoplasmic β-catenin levels.

The Wnt/β-catenin pathway plays a significant role in regulating cell proliferation, differentiation, and development [29-31]. Central to this pathway is the regulation of intracellular β-catenin levels. They are controlled by GSK-3β-mediated phosphorylation at Ser33/37/Thr41 in the β-catenin destruction complex, composed of APC, Axin, casein kinase 1, and GSK-3β [30]. Phosphorylated β-catenin is recognized by the β-transducin repeat-containing protein, an F-box E3 ubiquitin ligase, and degraded through a proteasomal-dependent pathway [31, 32]. It is noteworthy that MRBE still promoted β-catenin degradation in the presence of LiCl, a GSK-3β inhibitor, indicating that GSK-3β is not required for MRBE-induced β-catenin degradation. Previous studies reported that cyclin-dependent kinase 2/cyclin A or cyclin E and protein kinase Cα are also involved in β-catenin degradation [33, 34]. Thus, the activity of these kinases may be necessary for MRBE-mediated β-catenin degradation. Recently, ursolic acid has been shown to decrease the β-catenin levels by increasing the level of Wnt antagonists, sFRP4 and DKK1, in breast cancer stem-like cells [35].

Constitutive upregulation of the Wnt/β-catenin pathway frequently occurs in MM without the presence of mutations in this pathway [4]. In addition, the stimulation of the Wnt/β-catenin pathway by Wnt3a and LiCl significantly increases the proliferation of MM cells [36]. Therefore, suppressing this pathway might be an effective strategy for MM treatment. The small molecule PKF115-585 inhibits MM cell proliferation by disrupting the interaction of the transcriptionally active β-catenin/TCF complex [37]. Recently, ICG-001, which can inhibit the formation of the transcriptional coactivator CBP/β-catenin complex, was shown to selectively promote apoptosis in primary MM cells [38]. In this study, we demonstrated, for the first time, that MRBE and its constituent, ursolic acid, induces cell cycle arrest and apoptosis by suppressing the Wnt/ β-catenin pathway in multiple myeloma cells.

Based on one-point estimation with the standard compound, the molar concentration of ursolic acid in 40 μg/mL MRBE was 0.13 μM, which is lower than the minimal effective concentration on MM cell growth inhibition (Fig. S3). Several studies have reported that natural compounds isolated from MRBE inhibit the proliferation of MM cells. Betulinic acid induces apoptotsis and cell cycle arrest by inhibiting NF-kB pathway in multiple myeloma [39]. In addition, β-sitosterol promotes apoptosis through activating AMP-activated protein kinase (AMPK) and c-Jun N-terminal kinase (JNK) [39]. In this context, though the estimated concentration of ursolic acid in MRBE may not be sufficient for MRBE-mediated suppression of MM cell proliferation, ursolic acid in MRBE, in a synergy with betulinic acid and/or β-sitosterol, can inhibit the growth of MM cells.

In conclusion, this is the first study to identify the antiproliferative potency of MRBE and ursolic acid against MM cells and reveal the molecular mechanism. MRBE and ursolic acid accelerate oncogenic β-catenin turnover and repress β-catenin-dependent gene expression, thereby suppressing MM cell growth through apoptosis. Therefore, MRBE and ursolic acid have potential to be developed into preventive or therapeutic agents for MM treatment.

## Supplemental Materials

Supplementary data for this paper are available on-line only at http://jmb.or.kr.
