# Supplementary material for: Root Bark of Morus alba L. and Its Bioactive Ingredient, Ursolic Acid, Suppress the Proliferation of Multiple Myeloma Cells by Inhibiting Wnt/β-Catenin Pathway
Source: J Microbiol Biotechnol. 2021 Sep 25;31(11):1559–67. doi: 10.4014/jmb.2109.09002 (PMC9706038; doi:10.4014/jmb.2109.09002)
Supplement: Supplementary file 1 [file jmb-31-11-1559-supple.pdf]

J. Microbiol. Biotechnol.
https://doi.org/10.4014/jmb.2109.09002

Supplementary data

Table S1. Compound list

Compound	*RT (min)*	*MSMS similarity*	*Area*
2,3,5,6-Tetramethylpyrazine	6.286	97.8	23252576059.4
Pipecolic acid	1.557	88.2	16962426677.8
Adenosine	2.037	96.4	13092760204.6
Betaine	1.466	91.5	9765144346.1
9-Oxo-10(E),12(E)-octadecadienoic acid	22.112	91.5	8778321282.5
Esculin	7.136	88.3	7191291100.7
Scopoletin	7.405	89.6	6589184708.1
Choline	1.423	87.8	5816093510.1
Hexadecanamide	25.852	94	4802281361.2
D,L-Camphor	8.399	93.6	4539504142.0
L-Phenylalanine	3.565	96.1	4004785153.8
L-Isoleucine	2.328	85.7	3632272948.4
Indole-3-acrylic acid	6.476	93.3	3627603683.7
7-Hydroxycoumarine	7.002	94.7	3097087245.1
Luteolin	12.349	80.6	2877244051.6
4-Guanidinobutyric acid	1.897	91.9	2863865779.9
Proline	1.638	86.4	2252165470.6
L-Norleucine	2.194	84.5	2231210211.0
Esculetin	6.84	87.9	2014284782.1
DL-Arginine	1.399	90.9	1514149881.3
2-Hydroxycinnamic acid	2.096	88.2	1316662583.1
Glycerophospho-N-palmitoyl ethanolamine	21.336	75.3	1259469631.4
Morin	11.658	91.1	1152442324.5
Apigenin	13.412	95.4	1058778191.1
Prolylleucine	1.959	80.4	992470630.1
Quercetin-3β-D-glucoside	8.74	89.3	974293833.9
Valine	1.895	89.7	870666506.6
Sedanolide	14.078	74.4	852700142.4
Nicotinamide	1.907	92.4	812476208.6
Eriodictyol	8.336	75.1	765907026.5
5α-Dihydrotestosterone	22.918	81.8	749525529.9
2-Amino-1,3,4-octadecanetriol	19.756	73.4	749464263.5
Daidzein	23.061	85.3	749389562.3

Nicotinic acid	1.903	94.3	690434279.5
Butein	11.087	73	684289300.9
Osajin	19.175	74.7	655181530.9
Isoliquiritigenin	14.547	97.4	589212331.0
Maltol	5.271	92.4	509654616.6
N-(2,4-Dimethylphenyl)formamide	8.987	74.5	495202891.2
Asparagine	1.567	86.8	480269320.3
N-α-L-Acetyl-arginine	1.517	90.9	470380569.5
Formononetin	24.467	84.4	462091743.5
Ethyl oleate	23.867	85.5	456297249.0
Mono(2-ethylhexyl) phthalate (MEHP)	22.357	84.6	431506390.6
Thymine	2.272	85.5	409324195.0
Adenine	1.883	86.6	392298019.8
19-Nortestosterone	15.347	86.1	358119948.8
Ursolic acid	25.278	86.2	331350824.1
Taxifolin	8.704	87.8	318079047.9
(±)-Abscisic acid	6.665	82	312320913.6
DL-Stachydrine	2.125	85.1	308715206.7
2'-O-Methyladenosine	2.464	81.7	307468578.7
2,6-Di-tert-butyl-1,4-benzoquinone	16.263	70.2	282439446.1
Alternariol	10.334	74.8	272843113.9
3-Succinoylpyridine	6	71.7	259150576.9
Resveratrol	11.61	91.5	256913361.0
α-Linolenoyl ethanolamide	18.283	73	251212999.2
Piceatannol	18.624	81.8	239327352.3
4-Acetamidobutanoic acid	2.312	78.3	238796218.0
Coumarin	8.183	76.5	236230469.2
Vanillin	9.117	78	217152031.9
8-Hydroxyquinoline	10.657	89.5	198948175.7
D-Glucosamine	1.405	90.3	198922095.2
Pyridoxine	1.95475	86.9	194336849.5
Citrinin	13.703	71.5	194025789.1
Uracil	1.997	83.2	188221301.4
Oleamide	26.095	87.7	184056880.1
Kojic acid	5.543	86.4	180254958.3
Palmitoleic acid	25.888	86.7	174478300.6
Estriol	16.688	78.2	172035612.6
Vitexin	9.337	81.9	169889993.3

trans-Cinnamaldehyde	5.442	82.2	167977100.8
Methyl cinnamate	10.169	80.4	159884553.0
Naringenin	13.537	87	157819806.1
Hypoxanthine	2.118	83	149428042.3
Purpurin	11.052	79.4	149236385.0
3-Methoxybenzaldehyde	4.444	81.1	146455494.5
Norfentanyl	13.52	75.2	135939645.2
Isophorone	10.992	82.8	117000118.5
Isorhamnetin	12.016	84.2	115973391.9
Trigonelline	1.472	93.8	115942385.5
8Z,11Z,14Z-Eicosatrienoic acid	22.938	85.9	114726528.0
Kuromanin	10.409	82.1	112970307.4
Nootkatone	20.758	90.2	111785757.4
3,4-Dihydroxybenzaldehyde	9.58	85.9	107114123.4
Emodin	10.652	81.1	106612215.4
Oxymatrine	12.952	78.6	103455746.7
2,3-Dihydroxybenzoic acid	12.153	82.1	102202602.7
Cuminaldehyde	9.543	77.5	100338123.8
Apocynin	4.78	71.7	98155032.6
Guanine	1.883	88.4	96039704.0
18-β-Glycyrrhetinic acid	20.982	92.3	95085329.7
Polygodial	21.439	81.3	93530786.5
Kaempferol	13.681	91.8	91488538.5
6-Methylquinoline	6.477	73.8	82755522.6
Carvone	15.02	89.2	72194810.1
Biochanin A	13.8	72.4	71148967.2
2,6-Diethylaniline	8.691	70.2	67662060.3
2'-O-Methylguanosine	2.775	81.4	67386521.1
Chrysin	18.155	84.9	67152653.8
Glycitein	16.565	90	65693330.1
4-Hydroxybenzaldehyde	8.335	79.6	64739878.4
Chlorogenic acid	5.914	90.1	59539551.7
Icaritin	19.544	80.6	57693801.2
L-Histidine	1.325	90	56662496.7
Betulin	19.202	85.4	55867864.3
N-Acetyl-DL-tryptophan	10.179	71.6	52911934.2
Penicillic acid	6	71.7	48774418.7
4'-Methoxyacetophenone	7.769	77.8	48540550.1
4-Coumaric acid	9.238	84.2	47549325.2

Aflatoxin G1	12.294	78.2	46496795.2
Quercetin	11.065	78.8	41805137.1
Jasmonic acid	13.328	76.3	41743852.2
Arachidonic acid	23.729	79.9	39228029.0
Puerarin	7.82	93	38199100.8
Prunin	10.569	86.1	34734343.0
Acetophenone	8.847	70.9	27001052.7
Capsaicin	12.386	71.6	26277452.5
3-Acetyl-11-keto-β-boswellic acid	21.537	72.6	13182932.9
2-Hydroxybenzothiazole	20.48	85.3	1977624.8
Caprolactam	19.875	86	1777756.9
